# Engineering Thermoresponsive Biointerfaces Using Graft-To Strategies: Connecting Polymer Architecture to Temperature-Dependent Surface Wettability

**DOI:** 10.1021/acs.macromol.6c00545

**Published:** 2026-06-15

**Authors:** Kelly M. Bukovic, Steven R. Caliari, Rachel A. Letteri

**Affiliations:** † Department of Chemical Engineering, 118719 University of Virginia, Charlottesville, Virginia 22903, United States; ‡ Department of Biomedical Engineering, University of Virginia, Charlottesville, Virginia 22903, United States

## Abstract

Temperature-responsive substrates provide a promising strategy for addressing issues in biomaterials design, but their utility hinges on achieving predictable, tunable surface responses through deliberate thermoresponsive polymer design. Here, we investigate how varying polymer architecture, namely, surface attachment point density and chain length, influences wettability above and below the lower critical solution temperature (LCST) and the magnitude of temperature-driven contact angle change of polymer-grafted glass surfaces. Using random copolymers of thermoresponsive di­(ethylene glycol) methyl ether methacrylate (DEGMA) units and surface attachment point aminoethyl methacrylate (AEMA) units, we show that increasing polymer attachment point density from 2 to 30 mol % while holding the degree of polymerization constant decreases the magnitude of the temperature-driven contact angle change from 15.2 ± 0.5 to 5.7 ± 0.03°. This suggests that higher attachment point densities produce compact polymer loop structures on the surface that constrain thermoresponsive units and hinder hydration below the LCST. In contrast, increasing the degree of polymerization from 49 to 350 at a constant attachment point density increases the magnitude of the contact angle shift from 7.4 ± 4.4 to 22 ± 2.2°, likely attributed to enhanced chain mobility with increasing chain length. Notably, all surfaces converge to approximately the same contact angle above the LCST, regardless of polymer architecture, likely owing to the formation of similarly collapsed, dehydrated polymer layers that present comparable surface compositions to the interface. This work demonstrates that polymer architectures that allow greater chain mobility, achieved through lower attachment point density or longer chain length, produce larger thermal surface responses below the LCST, driven by enhanced hydration. By linking polymer loop structure and chain mobility to surface behavior, these insights provide a framework for designing biointerfaces with predictable and tunable properties.

## Graphical abstract

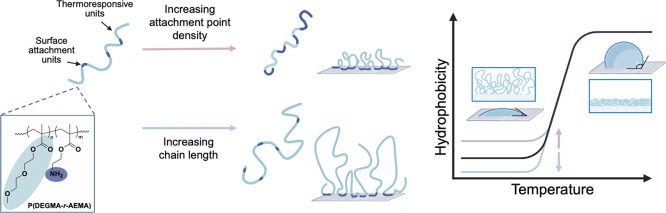

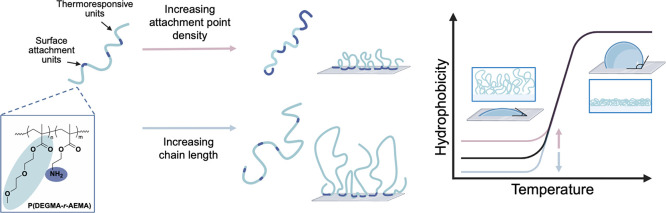

## 1 Introduction

“Smart” surfaces that change physicochemical properties in response to environmental stimuli are gaining traction in various fields, with temperature-responsive surfaces showing particular promise in addressing challenges in biomaterial design.^1–4^ When these surfaces exhibit sharp and reversible hydrophobicity changes near physiological temperature, they enable dynamic modulation of bioadhesive processes for applications such as protein chromatography,^5–7^ enzyme-free cell sheet harvest,^8–10^ and biosensor development.^11–13^ The functionality of these surfaces is typically achieved by tethering lower critical solution temperature (LCST)-type thermoresponsive polymers that are soluble via hydrogen bonding below the LCST, but become hydrophobic near physiological temperature.^14^

However, a critical gap remains in understanding how to systematically design thermoresponsive systems for specific biological contexts. While the coil-to-globule mechanism of LCST-type thermoresponsive polymers is well established, previous studies have highlighted that variables such as polymer composition, molecular weight, and grafting density impact cell adhesion and detachment^8,15^ as well as interactions with biomolecules.^6,16^ For example, increasing the length of grafted poly­(*N*-isopropylacrylamide) (PNIPAAm) brushes decreases the rate of cell adhesion at physiological temperature but accelerates the rate of detachment below the LCST,^8^ illustrating that a single architectural parameter can significantly alter cell-surface interactions. More broadly, this sensitivity to surface properties extends across biological systems: while one cell type or biomolecule can respond differently to surfaces with varying architectures, different cell types or biomolecules can exhibit distinct responses to the same thermoresponsive substrate.^16–18^ This variability introduces challenges in predicting biomolecule and cell release and limits the broad applicability of existing systems. Therefore, the ability to precisely tailor surface properties through polymer design is critical for optimizing the performance across a range of biological systems.

While studies in a broad range of materials, including membranes and catalytic systems, identify polymer architecture as a key factor in directing surface wettability,^19–23^ in thermoresponsive polymer systems, interfacial behavior is described not only by the static wettability of a surface, but also by the magnitude of temperature-induced wettability transition. Importantly, these hydrophobicity shifts are also largely dependent on polymer architecture. For example, graft-from thermoresponsive polymers with a block of hydrophobic units demonstrated significant contact angle changes (Δθ = 20.3°), whereas randomly distributed hydrophobic units yielded modest shifts (Δθ = 11°).^16^ Similarly, solutions of nanoparticles grafted with a higher density of polymer chains showed greater shifts in transmittance between temperatures than those grafted sparsely.^24^ These findings highlight polymer architecture as a key factor in dictating thermal response, motivating us to explore the impact of other architectural variables on thermoresponsive surface behavior toward improving the tunability and predictability of these systems.

In this work, we tether random copolymers composed of thermoresponsive diethylene glycol methyl ether methacrylate (DEGMA) units and surface attachment aminoethyl methacrylate (AEMA) units to glass surfaces using a graft-to strategy. Unlike PNIPAAm-based systems, which often exhibit thermal hysteresis due to intra- and interchain hydrogen bonding,^25,26^ PDEGMA-based films lack this hysteresis and may provide more predictable and reproducible surface responses.^27,28^ In addition, the graft-to approach allows us to robustly characterize the polymers prior to surface attachment, enabling clear correlations between polymer design and interfacial behavior. Here, we systematically vary surface attachment point density and chain length to direct the size and conformation of loop-like structures on surfaces that we anticipate will impact surface wettability and the extent of hydrophobic transition (Scheme 1). By constructing a library of polymers with different architectures, we reveal how these parameters can be tuned to control surface properties, expanding the design potential of thermoresponsive platforms.

**Scheme 1:**
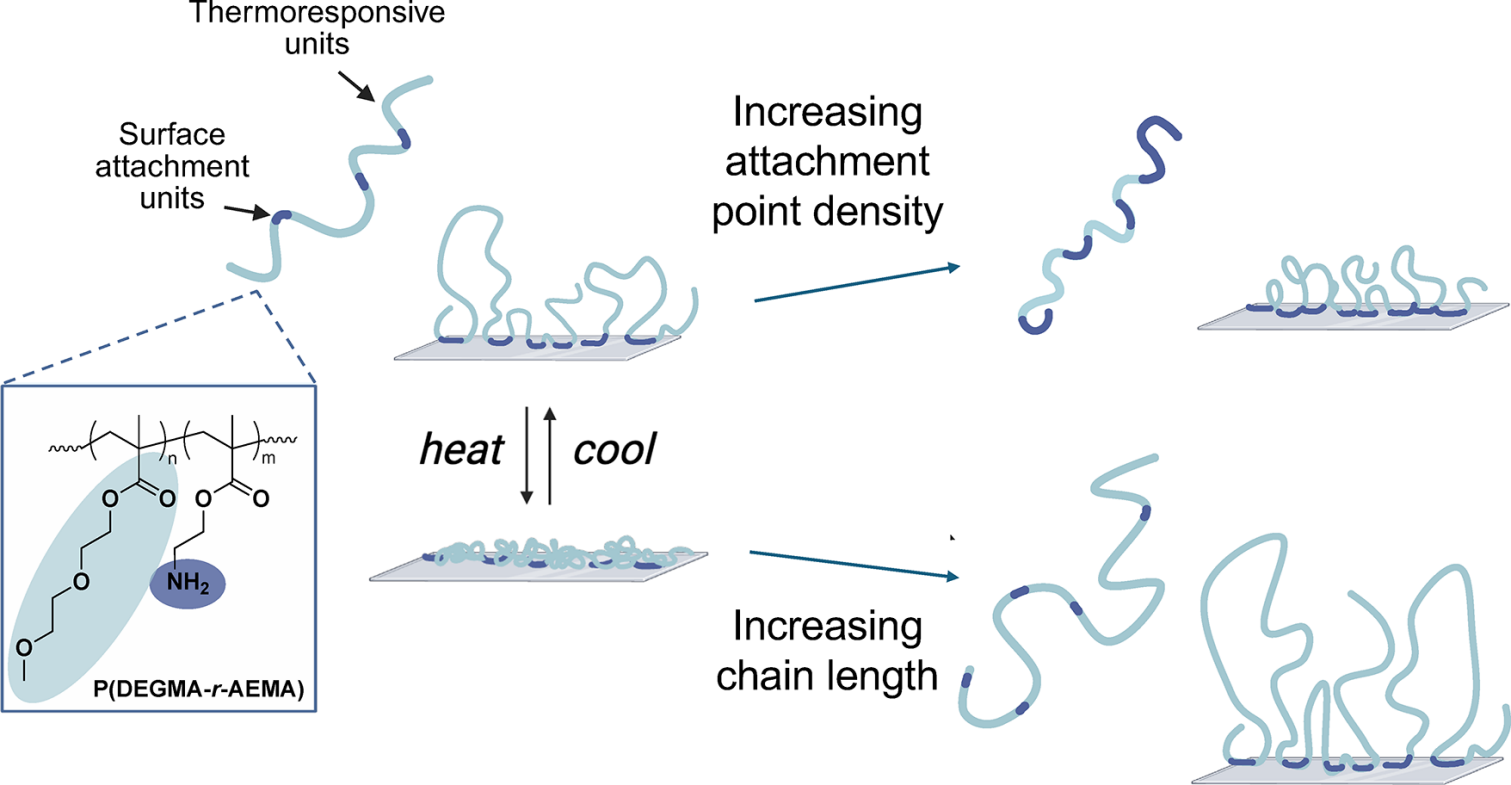
Schematic Demonstrating Graft-To Polymer Coatings Using P­(DEGMA-*r*-AEMA), Where AEMA Units Serve as Surface Attachment Points^a^

a Copolymers can be designed with varied surface attachment point density and chain length.

## 2 Results and Discussion

Because this work focuses on architectural differences between polymers, it was essential to have control over polymer molecular weight and composition. Thus, we used reversible addition–fragmentation chain transfer (RAFT) polymerization to synthesize our polymer libraries. Prior to synthesizing our polymer series, we performed a kinetics study to verify the intended random copolymer architecture (shown in Figure S1 and Table S1). Throughout this work, polymers are denoted PDRA-X-Y, where P­(DEGMA-*r*-AEMA) is abbreviated as PDRA, *X* indicates the target mol % of AEMA incorporated into the copolymer, and *Y* represents the target degree of polymerization (DP). We used a graft-to approach to attach the polymers to glass surfaces. Briefly, surfaces were functionalized using 3-aminopropyltriethoxysilane (APTES), and the resulting amines were immediately capped with glutaraldehyde (APTES-GA) to install pendant aldehyde groups for subsequent conjugation to AEMA units on the polymers via Schiff base formation. Following the conjugation reaction, sodium cyanoborohydride was used to stabilize the coatings via reductive amination (Scheme 2).^29^ Identical surface functionalization and polymer conjugation protocols were used to fabricate all polymer-coated surfaces to ensure observed differences in surface properties arose from variations in polymer architecture rather than differences in grafting conditions.

**Scheme 2:**
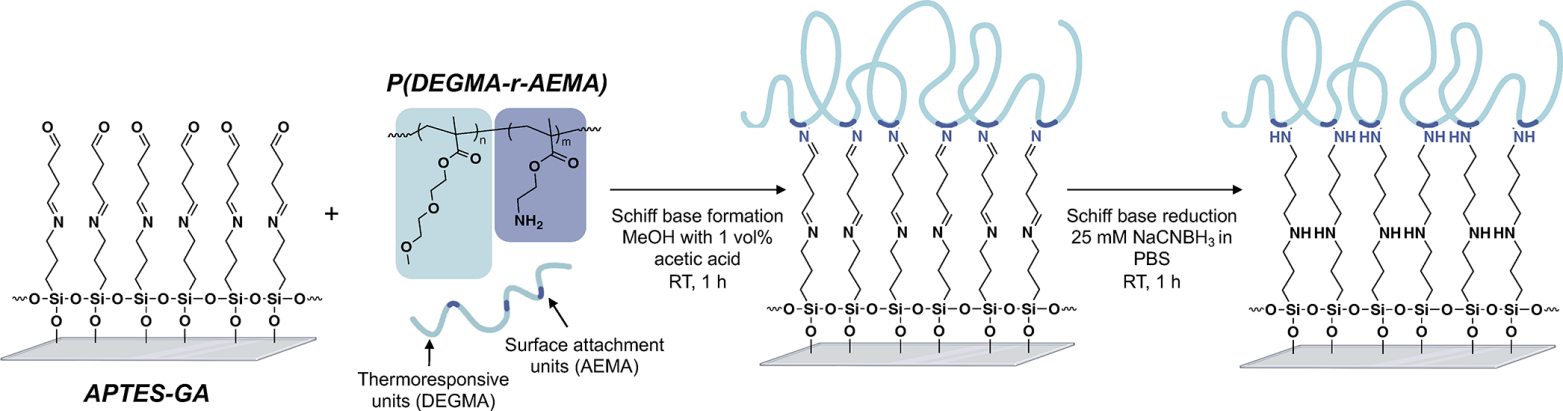
Attachment of Thermoresponsive Polymers to Borosilicate Glass Surfaces^a^

a Glass substrates were functionalized with APTES and subsequently capped with glutaraldehyde (APTES-GA) to generate pendant aldehyde groups for conjugation with amine functionalities on P­(DEGMA-*r*-AEMA) via Schiff base formation. Schiff bases were then reduced using sodium cyanoborohydride.

### 2.1 Synthesis of a P­(DEGMA-*r*-AEMA) Series with Increasing Attachment Point Density

Toward the goal of assessing the impact of attachment point density on thermoresponsive surface behavior, we synthesized a series of P­(DEGMA-*r*-AEMA) targeting 1, 5, 10, and 20 mol % AEMA. To isolate attachment point density, all polymers were synthesized with a target DP of 100 and reached similarly high conversions (>85%). Size exclusion chromatography (SEC) was used to ascertain the molecular weight (*M*_n_) and dispersity (Đ) of the polymer series, revealing that polymers were synthesized with similar chain lengths and Đ (1.35–1.38), allowing isolation of AEMA content as the primary variable (Figure 1, Table 1). Because low fractions of AEMA were below the quantifiable limits of ^1^H nuclear magnetic resonance (NMR) integration, the AEMA content in each polymer was quantified using a fluorescamine assay, with a calibration curve generated from AEMA standards to determine the mol % incorporation. Fluorescence intensities of polymer samples increased with increasing target mol % AEMA, demonstrating the achievement of increasing attachment point densities from 2 to 30 mol % within the series (Table 1).

**Figure 1:**
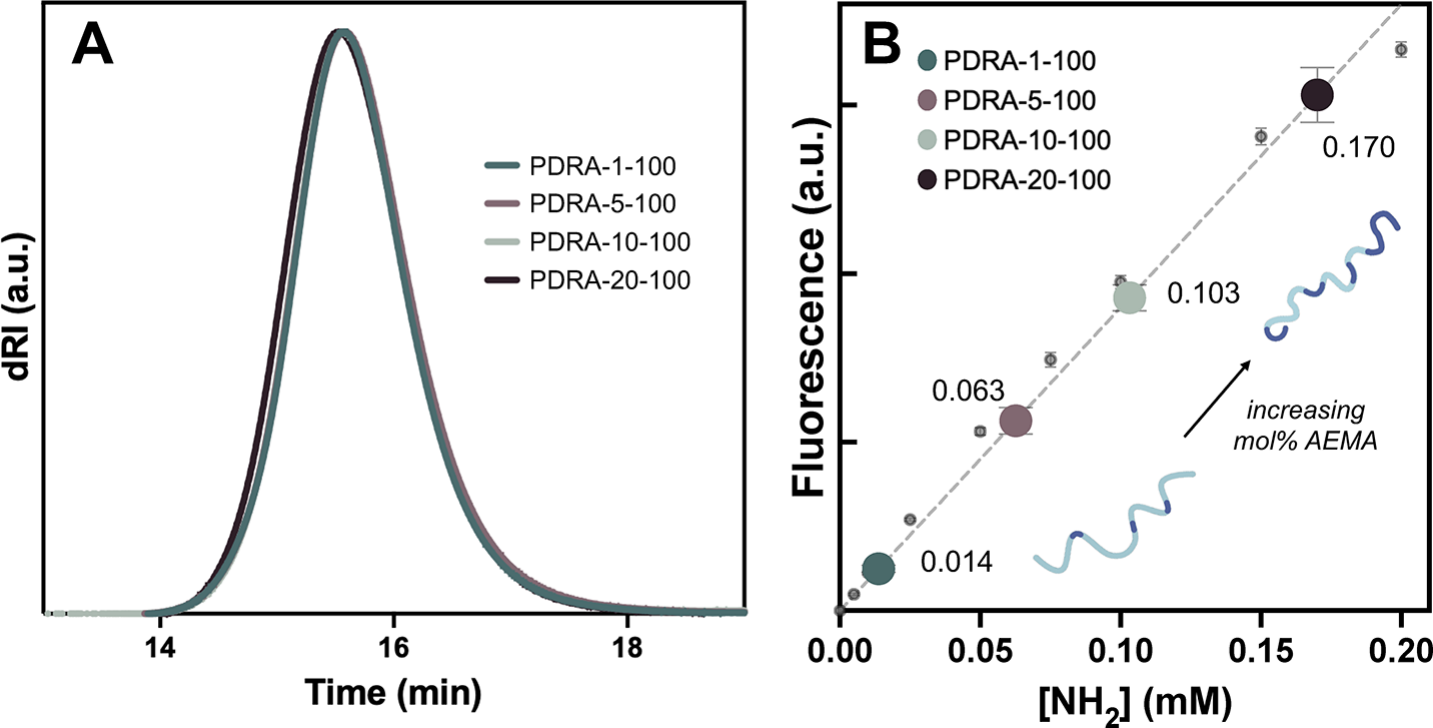
Characterization of PDRA-*X*-100 series. A) SEC traces of P­(DEGMA-*r*-AEMA) synthesized targeting 1, 5, 10, and 20 mol % AEMA (full traces shown in Figure S2). Overlapping peaks indicate similar molecular weights for all polymers in the series. B) Standard curve of AEMA generated by a fluorescamine assay, showing a linear relationship between the fluorescence intensity and the concentration of AEMA. Interpolated values for [NH_2_] for PDRA-1-100, PDRA-5-100, PDRA-10-100, and PDRA-20-100 are shown as the larger circles, indicating increasing incorporation of surface anchoring units. Values were interpolated from the mean of triplicate measurements per polymer.

**Table 1:** PDRA-*X*-100 Polymer Series Characterization Data

	Target mol % AEMA	mol % AEMA_exp_ ^a^	Target DP	DP_NMR_ ^b^	*M*_n_ (kDa)^c^	*Đ* ^c^
PDRA-1-100	1	2	100	97	28	1.36
PDRA-5-100	5	11	100	87	27	1.38
PDRA-10-100	10	18	100	88	28	1.35
PDRA-20-100	20	30	100	90	29	1.37

a Calculated from fluorescamine assay .

b Determined from ^1^H NMR spectroscopy conversion calculations (Figure S3).

c Determined by SEC analysis in trifluoroethanol with 0.02 M sodium trifluoroacetate, relative to poly­(methyl methacrylate) standards.

### 2.2 Surface Wettability Shifts as a Function of Polymer Attachment Point Density

After conjugating the polymers to glass substrates, surface wettability was assessed using static contact angle measurements (Figure 2) at 15 and 40 °C, which are below and above the reported LCST for PDEGMA (∼26 °C).^30,31^ Thus, we anticipate more hydrophilic surface behavior, reflected by lower contact angles, at 15 °C, and more hydrophobic behavior, reflected by higher contact angles, at 40 °C. To establish baseline surface behavior, we first measured contact angles on unfunctionalized glass, glass treated with APTES, and glass treated with APTES-GA. The APTES slides exhibited significantly higher contact angles than those of untreated glass, indicating the presence of the silane. After GA was added, we observed a slight decrease in contact angle, consistent with our previous observation.^29^ As expected, the control groups demonstrated constant contact angles at both 15 and 40 °C, indicating that temperature itself did not interfere with the measurements. To evaluate the thermal responsiveness, or magnitude of hydrophobic shift of each polymer-coated substrate, we compared changes in contact angle between 15 and 40 °C for each polymer-coated surface replicate (Figure 3).

**Figure 2:**
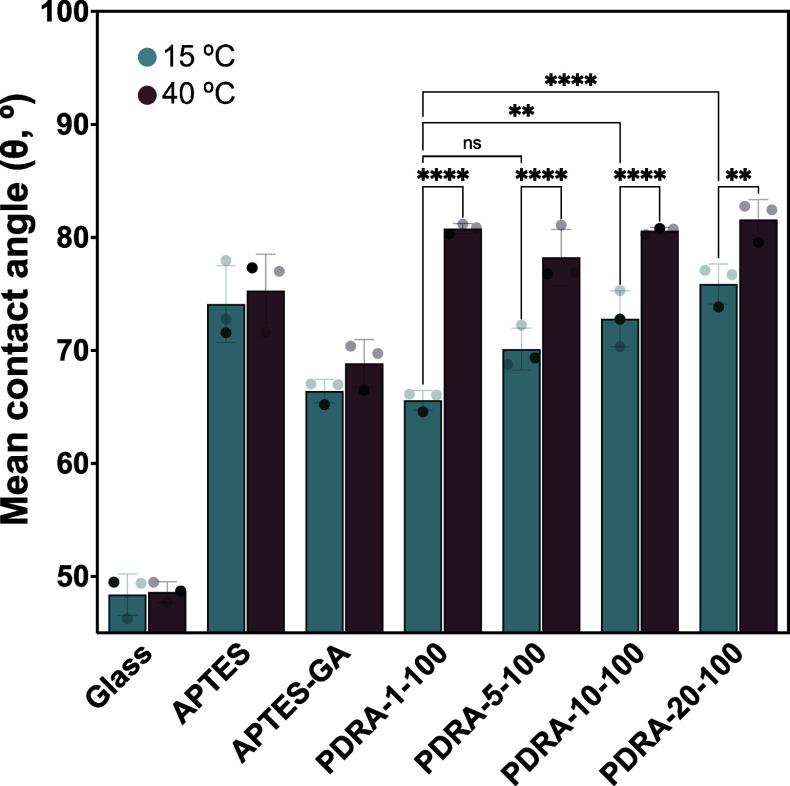
Static water contact angle measurements of polymer-coated glass substrates at 15 and 40 °C. Polymers with increasing attachment point density were grafted to APTES-GA functionalized surfaces. Contact angles increased with attachment point density at 15 °C, while measurements at 40 °C remained relatively constant across groups. Each point represents the average of three drops per surface ± SD; darkened points denote measurements derived from representative images shown in Figure S4. Statistical significance was determined using a two-way ANOVA with Tukey’s multiple comparisons test. **p < 0.01, ****p < 0.0001.

**Figure 3:**
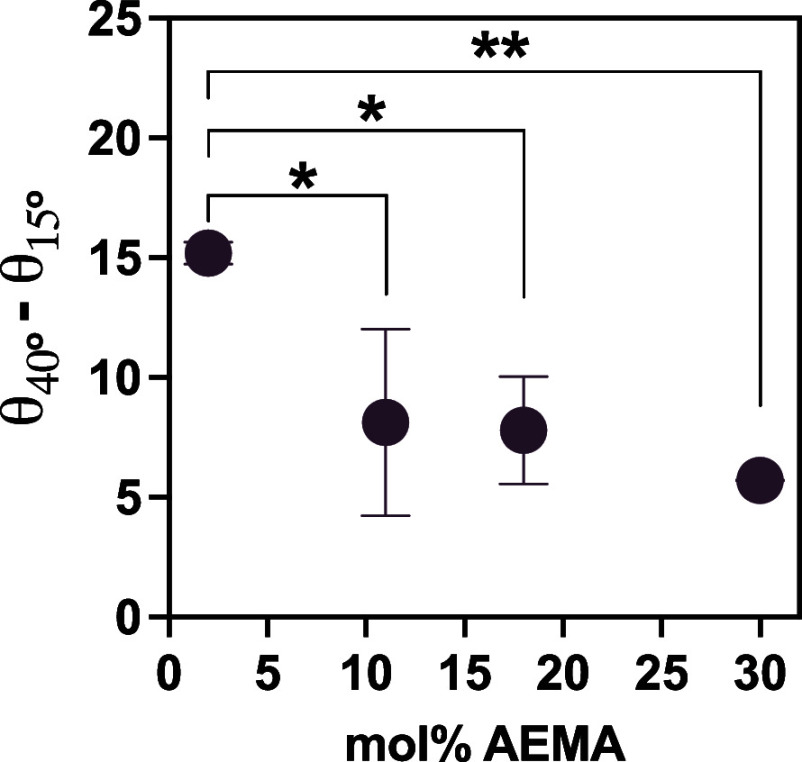
Contact angle value differences between 40 and 15 °C for each polymer-coated surface, calculated from the data in Figure 2. These changes show an inverse relationship between contact angle change and attachment point density, with larger differences demonstrated by polymers containing a lower density of attachment points (mol % AEMA). Each point represents triplicate substrate contact angle differences per condition ± SD. Statistical significance was determined using a one-way ANOVA with Tukey’s multiple comparisons test. *p < 0.05, **p < 0.01.

All polymer-coated surfaces exhibited thermoresponsive behavior; however, contact angles at 40 °C were relatively constant across the different polymers. Differences arose below the LCST at 15 °C, where contact angles increased with increasing attachment point density, correlating to more hydrophobic surface behavior. Contact angle differences between temperatures revealed an inverse relationship with attachment point density. Surfaces exhibited ∼15, 8, 7, and 6° differences between temperatures for polymers with 2, 11, 18, and 30 mol % AEMA incorporation (PDRA-1-100, PDRA-5-100, PDRA-10-100, PDRA-20-100), respectively.

We attribute the low-temperature changes in wettability to steric constraints imposed by polymer architecture: higher attachment point densities likely result in compact loops at the surface that restrict surface hydration below the LCST. In contrast, polymers with fewer attachment points retain greater chain mobility and longer segments of thermoresponsive units, promoting enhanced hydration and, thus, more hydrophilic surface behavior. Above the LCST, we suspect all polymer architectures adopt similar collapsed, dehydrated chain conformations, minimizing differences in surface wettability. This behavior mirrors observations in thermoresponsive hydrogel systems, where lower cross-link densities increase swelling, or hydrophilic behavior, below the LCST, owing to the conformational freedom of polymer chains.^32–34^ However, regardless of cross-link density, gels reach a similar polymer volume fraction in the collapsed state, reflecting comparable swelling behavior above the LCST.^32,34^ In the present work, varying surface attachment point density likely modulates network connectivity in a manner analogous to hydrogel cross-linking, influencing hydration-driven surface behavior below the LCST while leaving high-temperature wettability largely unchanged.

Together, these data suggest that polymer attachment point density not only influences surface wettability below the LCST but also directs the magnitude of thermal responsiveness, with excessive attachment to surfaces hindering the temperature-driven conformational changes required to generate substantial surface wettability differences. These results highlight attachment point density as a key design parameter for controlling the magnitude of the hydrophobic shift, expanding the tunability of thermally sensitive substrates.

### 2.3 Synthesis of a P­(DEGMA-*r*-AEMA) Series with Increasing Chain Length

Having identified mol % AEMA as a critical determinant in directing thermoresponsive behavior, we next investigated the impact of chain length on surface wettability and the magnitude of the hydrophobicity shift. To isolate this effect, we held the target AEMA content at 1 mol %, as this attachment point density produced the greatest thermal response, as demonstrated in Figure 3. Then, chain length was varied by targeting DPs of 50, 100, 250, and 500. The resulting chain length was assessed using SEC: elution times of polymer samples decreased with increasing target DP, showing that the series were synthesized with increasing chain length (Figure 4A). As the target DP increased, the achieved DP fell increasingly short of the target, with conversions decreasing from 98% for PDRA-1-50 to 70% for PDRA-1-500. Đ correspondingly increased with higher target DPs: 1.33, 1.39, 1.47, and 1.52 were achieved for PDRA-1-50, PDRA-1-100, PDRA-1-250, and PDRA-1-500, respectively. These trends are consistent with the inherent limitations of RAFT polymerizations at high molecular weight: reduced CTA concentrations can reduce the frequency of desired chain-transfer events and increase the potential for termination, limiting conversion and broadening molecular weight distributions.^35^ Nevertheless, the polymers synthesized here still exhibited increasing molecular weights, allowing us to assess the impact of chain length on surface wettability and thermal response. Further, the fluorescamine assay revealed similar fluorescent values for all samples, correlated to comparable AEMA content of ∼2 mol % (Figure 4B). This consistency in AEMA content ensures that observed differences in surface response can be attributed to chain length rather than variations in attachment point density.

**Figure 4:**
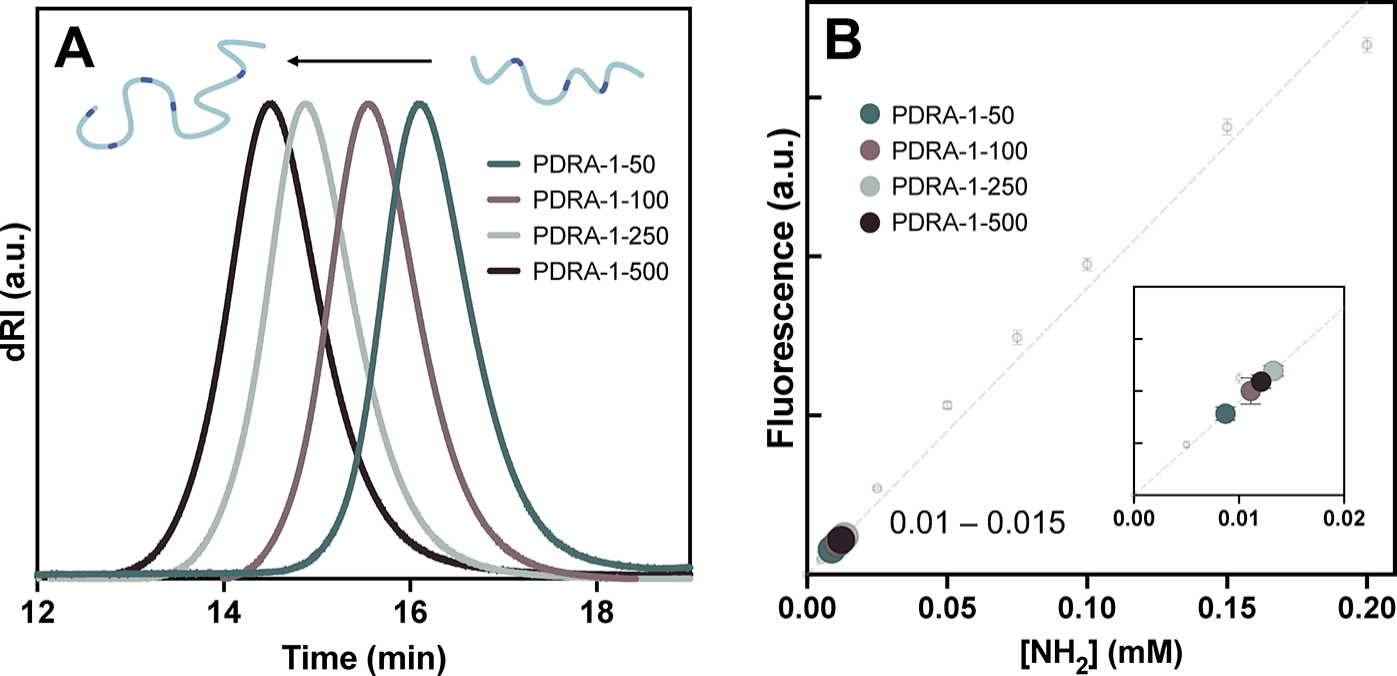
Characterization of the PDRA-1-*Y* series. A) SEC traces of P­(DEGMA-*r*-AEMA) synthesized targeting DP 50, 100, 250, and 500 (full traces shown in Figure S5). Peak maxima shifting left with increasing DP indicate increasing molecular weight of polymers. Eluent: trifluoroethanol with 0.02 M NaTFAc. Molecular weights were calculated relative to poly­(methyl methacrylate) standards. B) Standard curve of AEMA generated by a fluorescamine assay, showing a linear relationship between the fluorescence intensity and the concentration of AEMA. Interpolated values, shown as the larger points, for [NH_2_] for PDRA-1-50, PDRA-1-100, PDRA-1-250, and PDRA-1-500 were approximately 0.01–0.015 mM, demonstrating similar AEMA content for all polymers. Values were interpolated from the mean of triplicate measurements per polymer.

**2:** PDRA-1-*Y* Polymer Series Characterization Data

	Target mol % AEMA	mol % AEMA_exp_ ^a^	Target DP	DP_NMR_ ^b^	*M*_n_ (kDa)^c^	*Đ* ^c^
PDRA-1-50	1	2	50	49	16	1.33
PDRA-5-100	1	2	100	97	28	1.39
PDRA-10-250	1	2	250	220	54	1.47
PDRA-20-500	1	2	500	350	81	1.52

a Calculated from fluorescamine assay.

b Determined from ^1^H NMR spectroscopy conversion calculations (Figure S6).

c Determined by SEC analysis in trifluoroethanol with 0.02 M sodium trifluoroacetate, relative to poly­(methyl methacrylate) standards. PDRA-1-100 data are reproduced from Table 1.

### 2.4 Surface Wettability Shifts as a Function of Polymer Chain Length

After grafting the chain length polymer series to glass, contact angle measurements were conducted to evaluate surface behavior (Figure 5), with data from untreated glass, APTES, APTES-GA, and PDRA-1-100 groups reproduced from Figure 2. The thermal responses of each polymer-coated substrate are shown in Figure 6 as the change in contact angle between 15 and 40 °C as a function of polymer DP.

**Figure 5:**
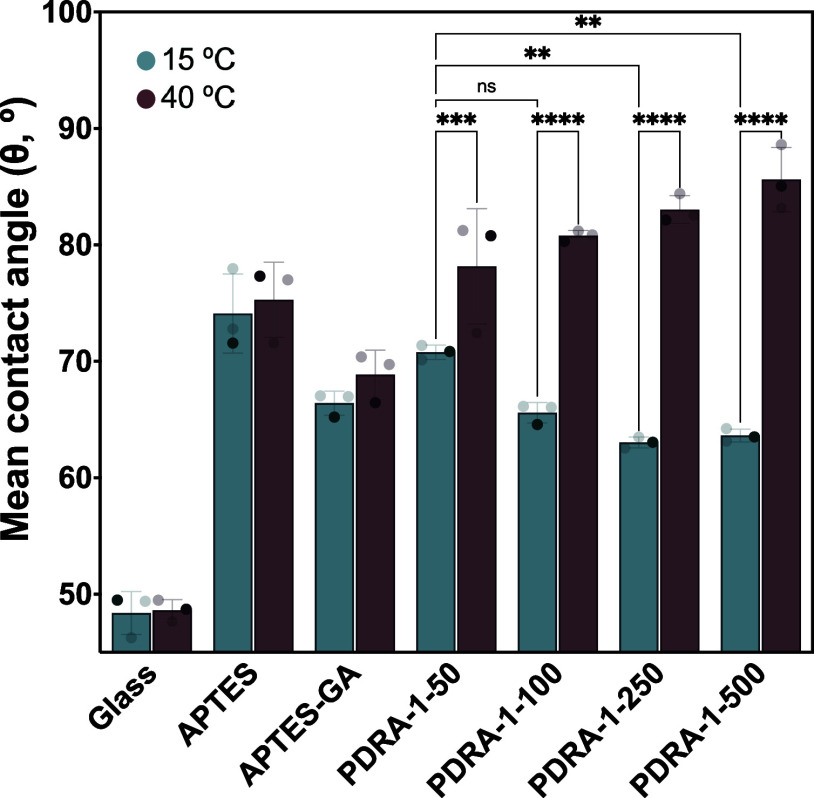
Static water contact angle measurements of polymer-coated glass substrates at 15 and 40 °C. Polymers with increasing chain length were grafted to APTES-GA functionalized surfaces. Contact angles decreased with increasing chain length at 15 °C, while measurements at 40 °C remained relatively constant across groups. Glass, APTES, APTES-GA, and PDRA-1-100 data are reproduced from Figure 2. Each point represents the average of three drops per surface ± SD; darkened points denote measurements derived from representative images shown in Figure S4. Statistical significance was determined using a two-way ANOVA with Tukey’s multiple comparisons test. **p < 0.01, ***p < 0.0005, ****p < 0.0001.

**Figure 6:**
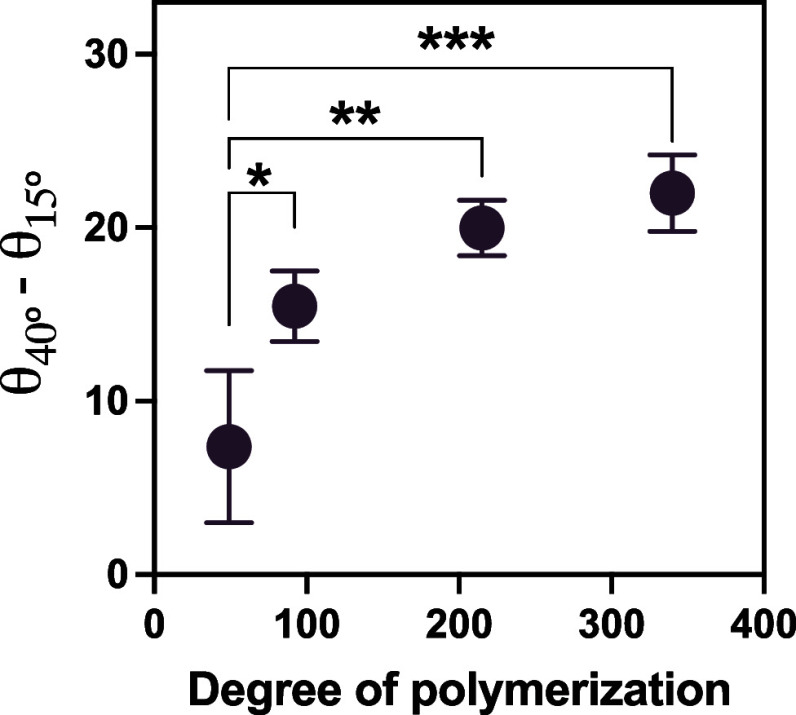
Contact angle value differences between 40 and 15 °C for each polymer-coated surface, calculated from data in Figure 5, revealing a direct relationship between contact angle change and chain length, with larger differences demonstrated by polymers of higher molecular weight (degree of polymerization). Each point represents triplicate substrate contact angle differences per condition ± SD. Statistical significance was determined using a one-way ANOVA with Tukey’s multiple comparisons test. *p < 0.05, **p < 0.01, ***p < 0.005.

Below the LCST, contact angles decreased with increasing chain length, but appeared to plateau at higher DPs. Similarly, we note comparable contact angles of all polymer surfaces above the LCST (40 °C). Nonetheless, increasing polymer chain length appeared to enhance the thermal response of polymer-coated substrates. Contact angle measurements revealed that substrates modified with PDRA-1-50 polymer exhibited a modest, temperature-driven contact angle change, with a difference of approximately 7° between temperatures. In contrast, polymers with greater DPs, PDRA-1-100, PDRA-1-250, and PDRA-1-500, produced progressively larger contact angle shifts of 15, 20, and 22°, respectively.

The decreasing contact angles below the LCST at higher DPs suggest that longer polymer chains have enhanced chain mobility compared with shorter chains, imbuing a greater capacity for temperature-driven conformational changes, whereas shorter chains are restricted, limiting hydration. These results are consistent with similar graft-from brush polymer systems, where increasing chain length was found to increase the rate of cell sheet detachment, likely attributed to the enhanced hydrophilicity of longer chains.^8^ However, we observed plateauing contact angles at higher DPs, with the PDRA-1-250 and PDRA-1-500 coatings displaying nearly identical contact angles below the LCST. This suggests that beyond a certain chain length, further increases may not significantly enhance the surface hydrophilicity. We posit that this phenomenon could arise from polymer chain entanglements: while the PDRA-1-500 polymers are more hydrophilic than PDRA-1-250 polymers on a per chain basis (i.e., more thermoresponsive units allow for greater uptake of water), larger chain lengths could increase interchain entanglement, particularly for loop-like structures.

Likewise, longer chain lengths may increase the density of collapsed polymer chains or the thickness of the collapsed film above the LCST, but the additional thermoresponsive units are likely buried within the layer, causing insignificant changes in surface chemistry experienced by the droplet. These results are consistent with a previous report that varied the thickness of a thermoresponsive poly­(*N*-isopropylacrylamide) (PNIPAAm) layer, finding that, while hydrophobicity increased with thickness, these increases did not scale linearly.^36^ In another study, dilute grafted PNIPAAm with shorter and longer brushes exhibited identical contact angles above the LCST, demonstrating that macroscopic surface behavior in the collapsed chain state was unaffected by brush length over the range tested.^37^

These data highlight that increasing the chain length provides a handle for enhancing the thermoresponsive behavior of polymer-coated substrates, primarily by increasing chain mobility and hydration below the LCST. However, this effect plateaus at higher DPs, suggesting that potential interchain entanglements limit further hydration by additional thermoresponsive units. Above the LCST, all surfaces exhibit similar contact angles regardless of molecular weight, indicating that the macroscopic wettability in the collapsed state is largely insensitive to the chain length. Despite this saturation, these findings illustrate the interplay between chain mobility and entanglement in controlling the temperature-dependent wettability, establishing design principles for engineering tunable thermoresponsive surfaces.

## 3 Conclusions

In summary, this work highlights polymer attachment point density and chain length as critical architectural parameters for controlling the wettability and thermal response of thermoresponsive polymer-grafted surfaces. Increasing the attachment point density breaks polymer chains into shorter segments, reducing the number of hydrophilic units per chain while constraining polymers into compact loops, suppressing the temperature-driven hydrophobic shift by limiting hydration below the LCST at the surface. Conversely, longer polymer chains with more consecutive hydrophilic units promote greater chain mobility and enhanced wettability transitions until a plateau in thermal response at higher molecular weights, potentially attributed to chain entanglements. Regardless of the polymer architecture, all surface contact angles converged to approximately the same value above the LCST. This suggests that the initial attachment of cells and biomolecules under physiological conditions may be minimally influenced by these architectural parameters, while detachment below the LCST is more likely to be governed by the architecture-dependent differences in the magnitude of the thermally induced interfacial transition. Although speculative, this suggests that these architectural parameters could enable the selective detachment of biomolecules or cells through tuned interfacial responses.

Together, these findings provide a design framework for fabricating polymer-coated interfaces with tunable surface behavior and thermal response. We envision that the platforms developed in this work will enable the rational optimization of substrates for cell sheet harvest, chromatography, biosensor development, or other applications in which precise control over interfacial properties is advantageous.

## 4 Instrumentation and Methods

### 4.1 Materials

Fisherbrand cover glasses (0.17 to 0.25 mm thickness, 18 mm × 18 mm) were purchased from Fisher Scientific. 3-Aminopropyltriethoxysilane (APTES, 99%), glutaraldehyde solution (GA, grade II, 25% in H_2_O), tetrahydrofuran (THF, ≥99.0%, ACS reagent, contains 200–400 ppm butylated hydroxytoluene (BHT) as inhibitor, suitable for HPLC), acetic acid (glacial, ACS reagent, ≥99.7%), 4,4′-Azobis­(4-cyanopentanoic acid) (ACVA, ≥98.0%), 2-[[(2-Carboxyethyl)­sulfanylthiocarbonyl]-sulfanyl]­propanoic acid (CTA, 95%), di­(ethylene glycol methyl ether methacrylate) (DEGMA, 95%), aminoethyl methacrylate hydrochloride (AEMA, contains ∼500 ppm phenothiazine as stabilizer, 90%), methanol (MeOH, ≥99.8%, ACS reagent, suitable for EPA 1613), trifluoroacetic acid (TFA, ≥99%), sodium cyanoborohydride (reagent grade, 95%), 2,2,2-trifluoroethanol (TFE, ≥99%), and sodium trifluoroacetate (NaTFAc, ≥98%) were purchased from Sigma-Aldrich. Dimethyl sulfoxide-d6 (DMSO-d6, D, 99.9%, reagent grade) was purchased from Cambridge Isotope Laboratories, Inc. A prepacked hydroquinone (HQ) and monomethyl ether hydroquinone (MEHQ) inhibitor removal column was purchased from Scientific Polymer Products, Inc. Ultrapure water (18.2 MΩ·cm) was obtained from a Thermo Scientific Smart2Pure water purification system.

### 4.2 Instrumentation

#### 4.2.1 Contact Angle Measurements

Sessile contact angle measurements of substrates were measured with a Krüss DSA25 goniometer equipped with a stage in a Peltier chamber for temperature control with a water bath. For experiments with thermoresponsive coatings, preliminary calibration established that setting the water bath to 8 °C produced a stable stage temperature of 15 °C, while a water bath temperature of 50 °C yielded a stage temperature of 40 °C, as measured by a temperature probe positioned directly on the stage. The stage was equilibrated at each temperature for 1 min before measurements were taken using 2 μL drops of ultrapure water. Three different positions on each sample were measured, with three measurements per drop taken 2 s apart. A total of 3 replicate surfaces per condition were tested.

#### 4.2.2 ^1^H Nuclear Magnetic Resonance (NMR) Spectroscopy

NMR spectroscopy was conducted on a 400 MHz Bruker Neo NMR spectrometer in DMSO-d6. Chemical shifts were referenced to the solvent residual peak at 2.5 ppm. Spectra were analyzed with MestReNova v14.3.2–32681.

#### 4.2.3 Size Exclusion Chromatography (SEC)

SEC was run in TFE with 0.02 M NaTFAc at a rate of 0.3 mL/min using a Tosoh system equipped with two isocratic pumps, a degasser, an autosampler, one 4.6 × 35 mm^2^ TSKgel Guard Super AW-H column (bead diameter: 9 μm), two 6 × 150 mm^2^ TSKgel Super AWM-H linear analytical columns (bead diameter: 9 μm), and a refractive index detector. Samples were prepared at 2 mg/mL with an injection volume of 20 μL. Number-average molecular weight *M*_*n*_ and dispersity *Đ* were determined relative to poly­(methyl methacrylate) (PMMA) standards.

#### 4.2.4 Fluorescence Measurements

Fluorescence measurements were performed using a Tecan Infinite 200 Pro microplate. Samples were excited at 382 nm, and the emission was collected at 475 nm.

### 4.3 Methods

#### 4.3.1 Coverslip Preparation

APTES and APTES-GA coverslips were fabricated at room temperature using an adapted protocol from van Deel et al.^38^ Glass coverslips were plasma-treated for 2 min at RT using a Harrick Plasma PDC-001 plasma cleaner on the high setting; then, APTES (100 μL) was applied to each surface. After 3 min, coverslips were rinsed with RO water 3 times and dried with Kimwipes. To functionalize surfaces with aldehyde groups, APTES-coated coverslips were incubated in a 5 vol % glutaraldehyde (GA) in water solution for 30 min in a glass Petri dish. Subsequently, the substrates were washed in RO water 3 times and dried with Kimwipes. We note that all APTES and APTES-GA substrates were modified and characterized within 24 h of fabrication due to the potential for hydrolysis of uncapped APTES and unreduced APTES-GA coatings in ambient conditions. For polymer-coated coverslips, polymer solutions (100 mg/mL) were prepared in methanol containing 1 vol % acetic acid. Polymer solution (50 μL) was applied to each APTES-GA-coated coverslip for 1 h. Coverslips were then rinsed in the methanol solution once, followed by RO water 3 times, and dried with Kimwipes. To stabilize the coatings, imine reduction was performed by incubating polymer-coated substrates with a 25 mM solution of sodium cyanoborohydride in PBS for 1 h. Coverslips were then rinsed in RO water, and excess water was removed by blotting with Kimwipes. We observed differences in contact angle measurements for substrates prepared under different humidity conditions, with samples fabricated during the summer generally exhibiting higher contact angles than those prepared in winter. Contact angles also varied somewhat with glass type and batch; however, the overall trends between experimental groups remained consistent across ambient conditions and substrate types.^29^

#### 4.3.2 Synthesis of P­(DEGMA-*r*-AEMA)

P­(DEGMA-*r*-AEMA) polymers were synthesized via reversible addition–fragmentation chain transfer (RAFT) polymerization. DEGMA, AEMA, CTA, and ACVA were added to a 20 mL scintillation vial, with reagent amounts for each copolymer listed in Table 3. Because the low amount of ACVA can be hard to accurately measure, a stock solution (10 mg/mL in 60/40 vol % THF/acetic acid) was prepared and added to the vial. DEGMA was purified by passage through a prepacked inhibitor removal column immediately before addition. Reagents were dissolved in 60/40 vol % THF/acetic acid at a monomer concentration of 2.5 M (2.1 mL). Initially, [acetic acid] was calculated equivalent to [NH_2_] in the highest mol % AEMA polymer; however, due to solubility issues, additional acetic acid was added until all reagents dissolved, resulting in a 40 vol % acetic acid solvent solution. The trithiocarbonate CTA and acidic solvent system were chosen to minimize aminolysis of the CTA by the primary amine of AEMA, as acidic conditions suppress nucleophilic attack. We note that while polymerizations were successful at 20 mol % AEMA, higher AEMA loading ratios were not compatible with the reaction conditions investigated. After reagents were dissolved, the solutions were degassed via bubbling with N_2(g)_ for 30 min and then placed in a heating block set to 70 °C for 24 h. An unpurified solution sample was taken for analysis by ^1^H NMR and SEC. The remaining reaction mixture was purified via dialysis using a dialysis membrane with a molecular weight cutoff (MWCO) of 3.5 kDa in a 4 L glass beaker with 3 solvent changes at least 4 h apart against 0.1% trifluoroacetic acid (TFA) in water. The purified polymer was lyophilized and stored at −20 °C until use.

**Table 3:** Reagent Amounts for RAFT Copolymerizations

	DEGMA		AEMA		**CTA**		**ACVA**	
	equiv	mmol (mL)	equiv	mmol (mg)	equiv	mmol (mg)	equiv	mmol (mg)
**PDRA-1**-**100**	99	5.2 (0.96)	1	0.05 (8.7)	1	0.05 (13.4)	0.1	0.005 (1.5)
**PDRA-5-100**	95	5.01 (0.92)	5	0.26 (43.7)	1	0.05 (13.4)	0.1	0.005 (1.5)
**PDRA-10-100**	90	4.77 (0.88)	10	0.53 (87.9)	1	0.05 (13.5)	0.1	0.005 (1.5)
**PDRA-20-100**	80	4.29 (0.79)	20	1.07 (177.6)	1	0.05 (13.7)	0.1	0.005 (1.5)
**PDRA-1-50**	99	5.13 (0.95)	1	0.05 (8.6)	2	0.1 (26.4)	0.2	0.01 (2.9)
**PDRA-1-250**	99	5.24 (0.97)	1	0.05 (8.8)	0.4	0.02 (5.4)	0.04	0.002 (0.6)
**PDRA-1**-**500**	99	5.25 (0.97)	1	0.05 (8.8)	0.2	0.01 (2.7)	0.02	0.001 (0.3)

#### 4.3.3 Quantification of AEMA Content in P­(DEGMA-*r*-AEMA)

To calculate the mol % AEMA in the polymers, a fluorescamine assay was used. A series of AEMA standards (0.005 mM to 0.15 mM) was prepared in 1X PBS. For the standards, 300 μL of each solution was transferred to a microcentrifuge tube, and fluorescamine solution (75 μL, 3 mg/mL in acetone) was added to each. For the polymer samples, polymer solution (20 μL, 2 mg/mL in PBS) and PBS (280 μL) were transferred to microcentrifuge tubes, and fluorescamine solution (75 μL) was added. Each standard/sample (100 μL) was pipetted into wells in a Nunc transparent 96-well plate in triplicate for each sample.

## Supplementary Material



## Notes

Copolymerization kinetics study; size exclusion chromatography (SEC) analysis of PDRA-X-100 series; ^1^H nuclear magnetic resonance (NMR) spectroscopy analysis of PDRA-X-100 series; contact angle images; SEC analysis of PDRA-1-*Y* series; ^1^H NMR spectroscopy analysis of PDRA-1-*Y* series (PDF)
