# Supplementary material for: Engineering Thermoresponsive Biointerfaces Using Graft-To Strategies: Connecting Polymer Architecture to Temperature-Dependent Surface Wettability
Source: Macromolecules. 2026 Jun 15;59(13):7972–80. doi: 10.1021/acs.macromol.6c00545 (PMC13374384; doi:10.1021/acs.macromol.6c00545)
Supplement: Supplementary file 1 [file ma6c00545_si_001.pdf]

Supporting Information

Engineering thermoresponsive biointerfaces using graft-to strategies: connecting polymer architecture to temperature-dependent surface wettability

Kelly M. Bukovic[1], Steven R. Caliari[1,2], Rachel A. Letteri[1]*

[1]Department of Chemical Engineering, University of Virginia, Charlottesville, VA, 22903, USA; [2]Department of Biomedical Engineering, University of Virginia, Charlottesville, VA, 22903, USA

*Corresponding author: Rachel A. Letteri, rl2qm@virginia.edu

Copolymerization kinetics study

Because polymer architecture is central to this work, we performed a kinetics study using an 90:10 DEGMA:AEMA comonomer ratio. We tracked corresponding monomer peak integrations over 24 h using ^{1}H NMR spectroscopy (**Figure S1, Table S1).** Constant integrations over time would indicate monomers were consumed at a similar rate, consistent with random incorporation of AEMA units along the polymer backbone. The RAFT polymerization was performed using conditions described in the main text for the PDRA-10-100 copolymer.

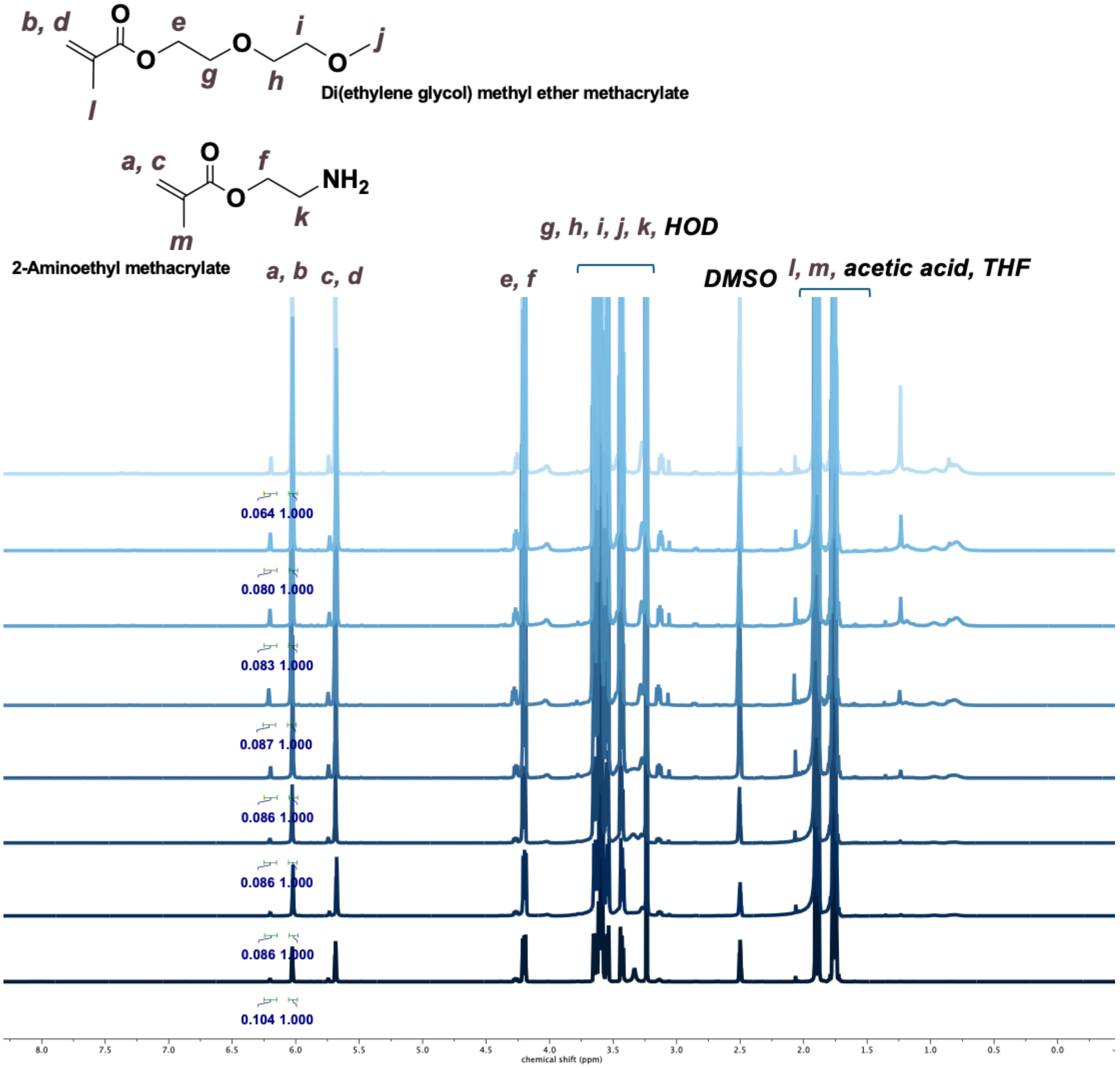

Figure S1. ^1H NMR spectra of 90:10 [AEMA]:[DEGMA] copolymerization over 24 h. Integrations were normalized to one proton on the DEGMA monomer.

Table S1. ^{1}H NMR monomer peak integrations monitored over time during RAFT polymerization.

Time (h)	∫a	∫b
0	0.104	1
0.5	0.086	1
1	0.086	1
2	0.086	1
4	0.087	1
8	0.083	1
12	0.080	1
24	0.064	1

^{1}H NMR integrations show relatively stable integrations over time, suggesting a copolymer architecture that aligns with attachment points distributed randomly rather than in a block-like sequence.

Size exclusion chromatography (SEC) analysis of PDRA-X-100 series

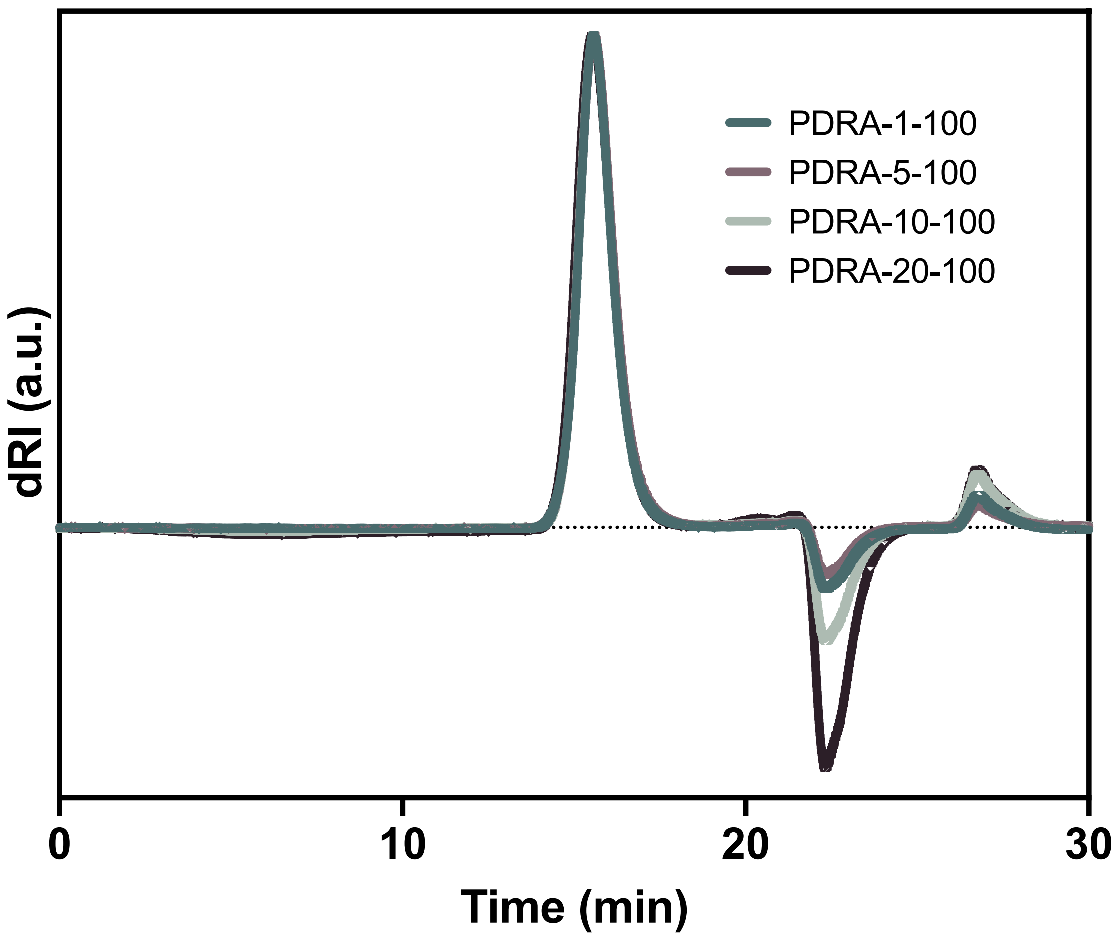

Figure S2. SEC traces of purified PDRA-1-100, PDRA-5-100, PDRA-10-100, and PDRA-20-100. Traces are normalized to peaks corresponding to polymer. Peaks eluting after 20 min are attributed to (and present in) the eluent. Eluent: Trifluoroethanol with 0.02 M NaTFAc. Molecular weights calculated relative to poly(methyl methacrylate) standards.

^{1}H nuclear magnetic resonance (NMR) spectroscopy analysis of PDRA-X-100 series

Polymer conversion was calculated from the relative integrations of the monomer and polymer peaks in the ^{1}H NMR spectrum of unpurified polymer solutions (**Figure S3**). Specifically, the signal of one vinyl proton on the DEGMA monomer (b, δ ≈ 5.6–5.7 ppm) was compared to the two methylene protons adjacent to the ester on the polymer (c', δ ≈ 3.90–4.15 ppm) and conversion was calculated as the fraction of one polymer proton peak integration over the sum of polymer and monomer peak integrations ($\frac{\int \frac{c'}{2}}{\int \frac{c'}{2} + \int b}$). Because smaller fractions of AEMA are below the quantifiable limit of NMR, it was

disregarded in conversion calculations for PDRA-1-100, PDRA-5-100 and PDRA-10-100. AEMA conversion was calculated for PDRA-20-100, as peaks corresponding the AEMA repeat units on the polymer were visible. AEMA conversion was calculated using the peak integration corresponding to one vinyl proton on the AEMA monomer (j, δ ≈ 5.72 – 5.76 ppm) compared to the integration of methylene protons adjacent to the primary amine on the AEMA polymer repeat units (l', δ ≈ 2.90 – 3.15 ppm), with monomer proton integrations (l, δ ≈ 2.90 – 3.15 ppm) subtracted. Unreacted CTA and ACVA were excluded from spectra labeling due to their negligible peak sizes in all polymer spectra.

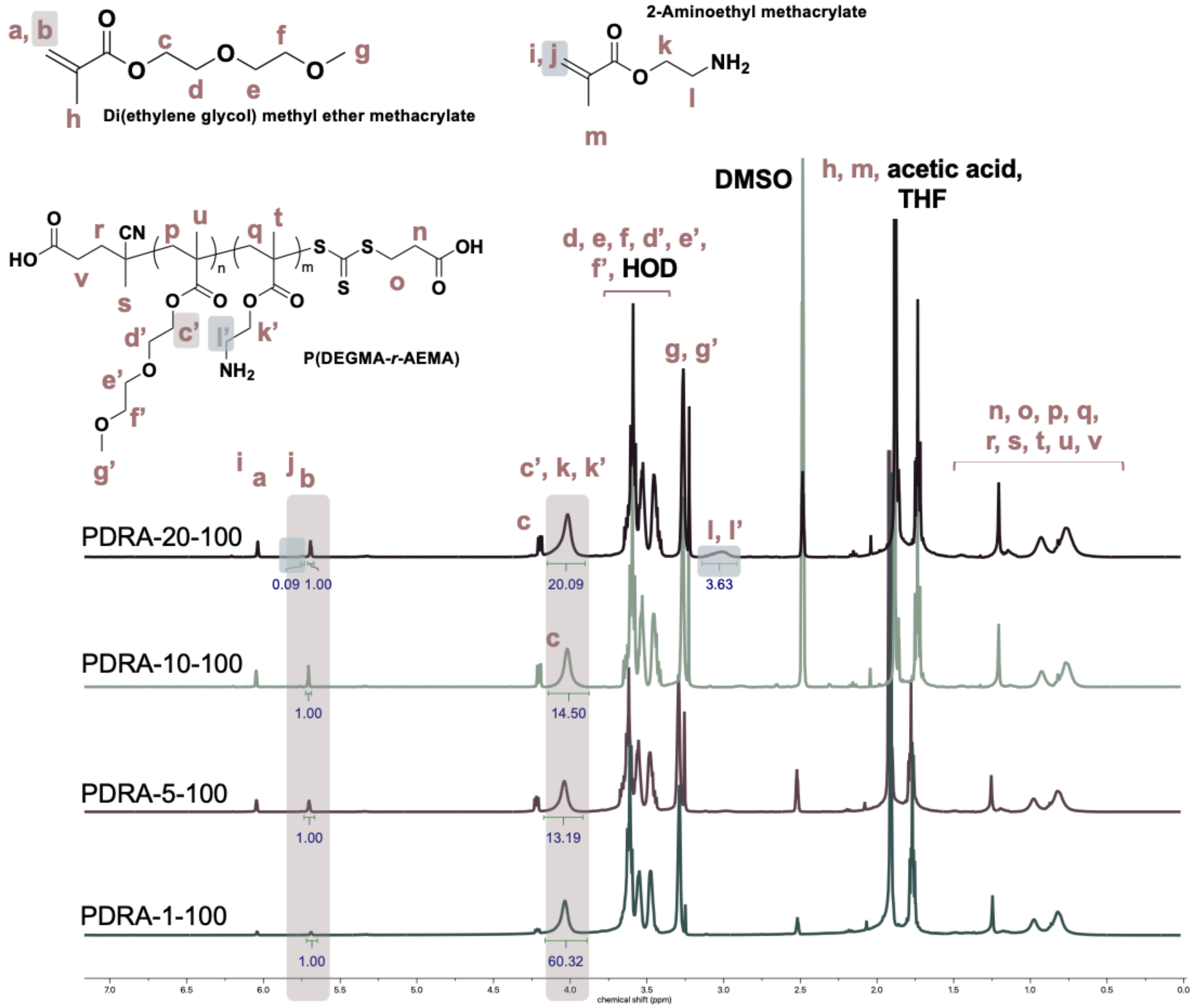

Figure S3. ^{1}H NMR spectra of unpurified attachment point density polymer series solutions with integrations used to calculate values shown in **Table 1**.

Contact angle images

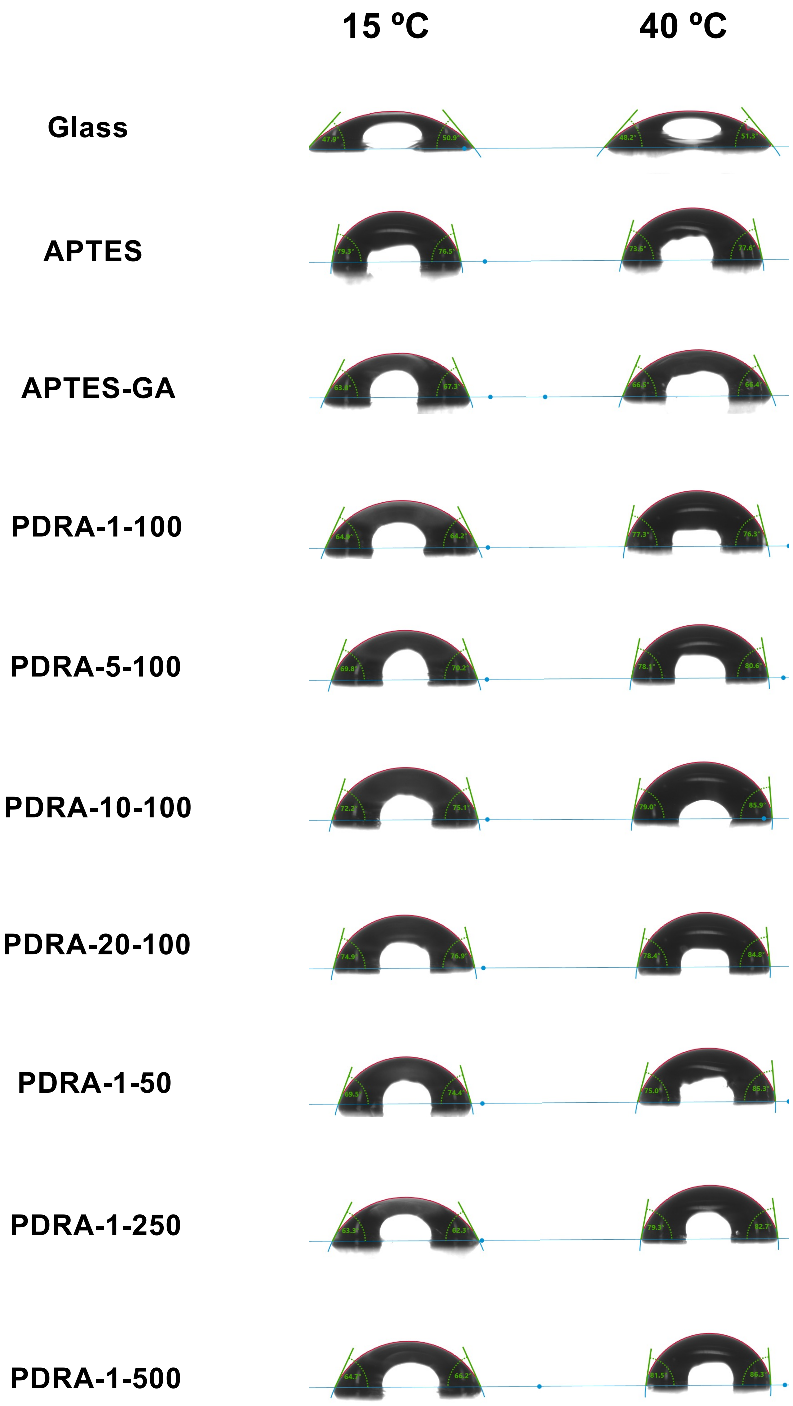

Figure S4. Representative sessile drop contact angle images of 2 μL ultrapure water droplets on each substrate used to derive the measurements shown in **Figures 2 and 5**. Images were acquired using Krüss ADVANCE software. Contact angles were determined using the tangent method; left and right angles are shown on each droplet image. Mean contact angle values (average of left and right angles shown in the images) were used for all reported data and analysis. Each image corresponds to one of nine contact angle images from one surface replicate used to calculate the darkened data point.

SEC analysis of PDRA-1-Y series

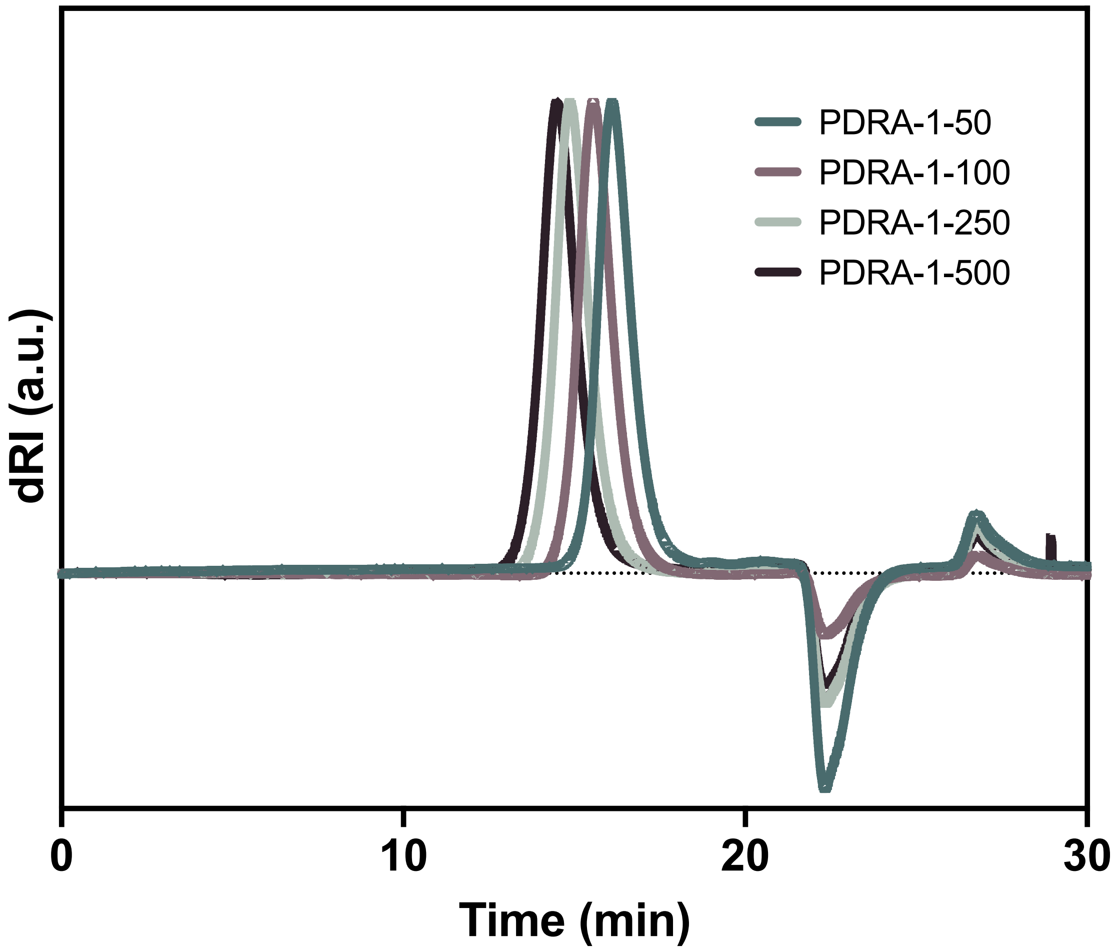

Figure S5. SEC traces of purified PDRA-1-50, PDRA-1-100, PDRA-1-250, and PDRA-1-500. Traces are normalized to peaks corresponding to polymer. Peaks eluting after 20 min are attributed to (and present in) the eluent. Eluent: Trifluoroethanol with 0.02 M NaTFAc. Molecular weights calculated relative to poly(methyl methacrylate) standards.

^{1}H NMR spectroscopy analysis of PDRA-1-Y series

Polymer conversion calculations were conducted identically to those described in **Figure S2**. Because the target AEMA content was held at 1 mol% for this polymer series, integration contributions by AEMA protons were disregarded and AEMA was excluded from spectra labeling.

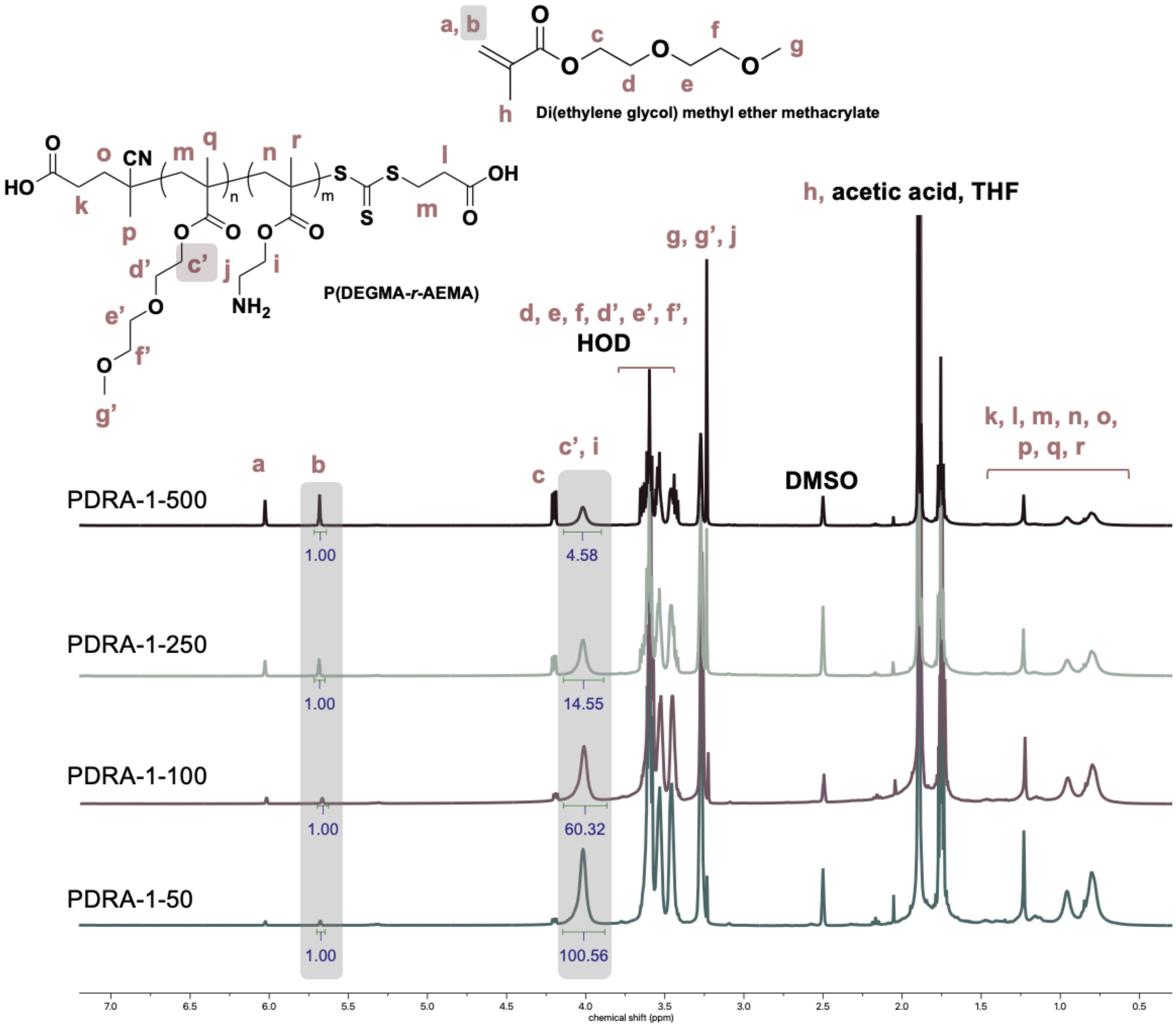

Figure S6. ^{1}H NMR spectra of unpurified chain length polymer series solutions with integrations used to calculate values shown in **Table 2**.
